# Extreme overvalued beliefs and identities: revisiting the drivers of violent extremism

**DOI:** 10.3389/fpsyg.2025.1556919

**Published:** 2025-03-05

**Authors:** Kolbrun Kristinsdottir, Julia Ebner, Harvey Whitehouse

**Affiliations:** ^1^Centre of Psychiatry and Mental Health, Wolfson Institute of Population Health, Queen Mary University of London, Charterhouse Square, London, United Kingdom; ^2^Centre for the Study of Social Cohesion, School of Anthropology and Museum Ethnography, University of Oxford, Oxford, United Kingdom; ^3^Calleva Centre of Evolution and Human Sciences, University of Oxford, Magdalen College, Oxford, United Kingdom

**Keywords:** identity fusion, fixation, extreme overvalued beliefs, violent extremism, lone-actor violence, threat assessment, terrorism

## Abstract

Recent efforts to understand violent extremism have appealed to the concept of *extreme overvalued beliefs* as a way of explaining fixation and extremist commitments. Extreme overvalued beliefs refer to an ego-syntonic fixation that grows more intense, absolute and emotional over time and is shared with a sub-community. However, while extreme overvalued beliefs precede many targeted attacks, most people who hold them do not resort to violence. Previous research has highlighted four ‘ingredients’ associated with an increased risk of violent extremism, only three of which are captured in studies linking extreme overvalued beliefs to violence: perceived outgroup threat, demonization of the outgroup, and endorsement of violence. We argue that the fourth element—missing from the literature on extreme overvalued beliefs—is identity fusion: a visceral sense of oneness with the group in which personal and group identities become functionally equivalent. The goal of this paper is to improve current understanding of the circumstances where individuals with extreme overvalued beliefs turn into potential attackers. We show that when certain types of extreme overvalued beliefs are combined with identity fusion it can lead to violent self-sacrifice. Drawing on evidence from psychiatry, evolutionary anthropology, behavioural psychology and computational linguistics, along with a forensic analysis of three high-profile case studies of lone-actor grievance-fuelled violence, we explore the interplay of these risk factors and propose a more encompassing construct for explaining violent extremism. We call this hybrid framework *Extreme Overvalued Beliefs and Identities* (EOBI), synthesising the findings of interdisciplinary research on pathological fixation and identity fusion.

## Introduction

Acts of extreme violence and self-sacrifice in the name of shared beliefs or ideologies have long perplexed clinicians, scholars, and the public alike. These acts can seem random and bewildering to observers but, in the mind of the perpetrator, are meticulously planned and justified. Building on decades of research in psychiatry, the concept of *extreme overvalued beliefs (EOBs)* has been introduced to strengthen the professional toolkit when assessing at-risk fixated individuals who might engage in targeted violence or acts of violent extremism (McKenna, 1984; Jaspers, 1997; Veale, 2002; McHugh, 2006; Rahman et al., 2019).^1^ EOBs are described as a cognitive-affective driver of fixation that is shared with a wider ingroup and cannot be clinically categorised as either delusions or obsessions (Rahman et al., 2020; Meloy and Rahman, 2021). A key question addressed in this paper is why some individuals who have EOBs are willing to commit acts of violence that jeopardise their relationships, finances, and even life and limb, while others who also exhibit EOBs are not. Answering this question would be of interest to several professional fields, including law enforcement, intelligence and security agencies, mental health services, and numerous academic disciplines such as sociology, anthropology, psychology, and medicine (Cooke and Logan, 2021; Logan et al., 2023).

We argue here that while EOBs provide an important framework for analysing the threat of violent extremism, the concept is insufficient to fully explain an individual’s shift from ideological extremism to violent action. As we will argue based on a review of the literature, EOBs do not inherently increase the risk of violence. Still, when they possess certain characteristics and generate additional risk factors, we expect the risk of violence to increase significantly. Specifically, our paper presents an extended conceptual framework that connects evidence from forensic psychiatry, social psychology and cognitive anthropology. Our integrated framework suggests that the risk of violence for individuals is highest when they (A) hold EOBs, which (B) are capable of producing ‘identity fusion’ (or short, fusion), and (C) involve perceptions of an existential threat to the ingroup, systematic demonization and/or dehumanisation of an outgroup and norms that condone, justify or glorify violence. Fusion produces a visceral feeling of oneness with the group, associated with a synergistic relationship between personal and group identities (Swann et al., 2012) and has been shown to significantly increase the risk of extreme pro-group violence and even self-sacrifice in defence of the group against real or imagined threats (Swann and Buhrmester, 2015). EOBs tend to be egosyntonic – in line with one’s values and personality – and shared with an ingroup, leading to notable changes in identity. Thereby, the proposed framework captures commonalities across interdisciplinary fields to improve the current understanding of violent extremism.

The fusion-violence link has been observed in empirical studies with diverse groups, including Libyan revolutionary battalions (Whitehouse et al., 2014), Cameroonian herders and farmers (Buhrmester et al., 2020), Indonesian religious fundamentalists (Kavanagh et al., 2020), British and Brazilian football hooligans (Newson, 2019), and captured fighters from Islamic State and Kurds on the battle frontlines (Atran, 2016). Recent computational linguistic analysis of the manifestos of lone-actor terrorists showed that, despite acting alone, these perpetrators were highly fused with their ingroups, which distinguished them from their non-violent extremist counterparts (Ebner et al., 2024). In addition to identity fusion, the study highlighted three other statistically significant patterns, including the perpetrator’s fixation on ideas of existential threat, demonised and/or dehumanised outgroups, and violence as a viable solution, which can all be interpreted as specific manifestations of EOBs. Identity fusion is also particularly relevant to the study of fixation and EOBs due to the crucial role of shared ego-syntonic beliefs and the subsequent changes to a person’s identity. Bridging previous research on EOBs and fusion, we believe our proposed integrated framework can provide valuable insight into the drivers and risk factors of violent extremism (Rahman et al., 2016, 2021; Ebner et al., 2022a, 2024). We will call our framework *Extreme Overvalued Beliefs and Identities (EOBI)*, as it adds extreme identity processes as the missing piece in the relationship between EOBs and violent extremism. To elucidate our EOBI framework, we will analyse three case studies of lone-actor grievance-fuelled violence. Based on these case studies that examine acts of extreme violence carried out by lone actors, we will review the commonly overlooked relationship between EOBs and identity fusion in providing the toxic combination of ingredients that can lead to a deadly mindset.

### The evolution of threat and threat assessment

The nature of terrorism has changed drastically in recent years (Fein, 2023). Expanding networks of online extremist subcultures have been accompanied by growing numbers of individuals with overbearing, overvalued, and extreme ideas (Wu and Ye, 2021). Governments and security professionals have introduced terms such as “mixed, unclear and unstable” or “salad bar extremism” to capture new forms of ideologically fluid violent extremism (The Soufan Center, 2021; Meier, 2023). Rising levels of polarisation, misinformation and conspiratorial thinking in the aftermath of the COVID-19 pandemic have contributed to this trend (Levinsson et al., 2021; Serdenes et al., 2023; Van de Cruys et al., 2023), which now poses a significant threat to public health and safety (Federal Bureau of Investigation, 2019; Levinsson et al., 2021; Holoyda, 2022).

This evolving threat needs to be addressed in the current technological climate, where social media serves as a breeding ground for fringe, violent, and abnormal beliefs (Rahman et al., 2016; Hoffman et al., 2020; Sarteschi, 2021; Holoyda, 2022). Online subcommunities based on conspiracy myths (such as QAnon, PizzaGate, the Great Reset and the Great Replacement), as well as extreme beliefs rooted in shared grievances and beliefs about ingroup purity and superiority (e.g., white supremacy, jihadism, anti-LGBTQA+, and Incels), have presented a significant challenge to policymakers (Wynia et al., 2017; Rahman et al., 2021; Broyd et al., 2022; Mulay et al., 2023). Not only can these ideologically extreme networks inspire the beliefs of lone-actor terrorists, but they are also a growing cause of deterioration in the social welfare and occupational functioning of young people insofar as they are associated with participation in hate speech and bigotry, adherence to violent ideologies, and extreme political or religious views, all of which contribute to social isolation (Neo et al., 2017; Alfano et al., 2018; Rahman, 2018; Pierre, 2019; Rahman et al., 2021; Sarteschi, 2021; Broyd et al., 2022; Tastenhoye et al., 2022; Mulay et al., 2023).

Research findings consistently reveal that grievance-fuelled lone terrorists share similar personality profiles, pathways to extremism, and grievances as other violent lone actors but differ significantly from group actors (Dhumad et al., 2022). In light of this, researchers have introduced the overarching category of *lone-actor grievance-fuelled violence* (LAGFV) (Ebbrecht and Lindekilde, 2023; Pathé et al., 2018). LAGFV is an umbrella term that captures multiple forms of targeted violence perpetrated by lone offenders, including school and workplace shooters, lone-actor terrorists, and targeted attackers motivated by a violent and grievance-fuelled ideology (Capellan, 2015; Clemmow et al., 2022; Ebbrecht and Lindekilde, 2023). Over the past decade, forensic psychiatry and psychology have relied heavily on structured professional judgment tools (SPJT) to assess the risk of individuals resorting to extreme forms of violence. While their empirical and theoretical support is varied (Scarcella et al., 2016), they predominately focus on four key components: ideology, affiliation, grievance and emotion (Kernot et al., 2022). Two widely used tools to assess the risk of lone-actor terrorism and targeted attacks include the Violent Extremist Risk Assessment (VERA-2r) (Pressman, 2009) and Terrorist Radicalization Assessment Protocol-18 (TRAP-18) (Meloy, 2018). Both tools have been used to explore various acts of extremist violence, including attacks by self-identified Incels (Collins and Clark, 2021; Chan, 2023) and individuals who partook in the Capitol riots (Challacombe and Patrick, 2023).

Maintaining validity and reliability across these SPJTs can be difficult as no clinically useful profile or cluster of risk factors has been identified for individuals more likely to commit ideologically motivated violence if they do not already adhere to extremist organisations (Wynia et al., 2017). The psychology of violent extremists has been widely studied, exploring the role of various mental health problems (Gill et al., 2021), psychiatric disorders (Sarma et al., 2022), and a range of psychological difficulties and vulnerabilities (Vermeulen et al., 2022). Despite this focus, many people have extreme views on particular subjects but do not pose any risk to themselves or others, which makes the identification and evaluation of violent extremism challenging and prone to false positives (Meloy and Gill, 2016; Meloy, 2018; Clemmow et al., 2022). The focus on interdisciplinary approaches within threat assessment and conceptual understanding of violent extremism has largely diminished in recent years, shifting the focus predominantly to individual vulnerabilities. While positive developments have been made for these data-driven approaches and SPJT used to identify at-risk individuals, it has been argued that this may be at the expense of theoretically driven explorations (Gill and Rottweiler, 2023). Importantly, while the violent act itself may be perpetrated by an individual, group dynamics and shared identity often play a significant role in the motivation and subsequent decision to attack alone (Gill et al., 2014; Ebner et al., 2022a). If it is recognised that group alignment does play a role, why are pre-existing theories and evidence from social psychology and evolutionary anthropology overlooked in modern threat assessment?

### Pathological fixation and extreme overvalued beliefs

Beliefs and ideologies associated with terrorist attacks are often preceded by lengthy periods of reflection and rumination, resulting in a mindset that provides elaborated justifications for violence (Borum, 2015). Therefore, in the context of violent extremism, encompassing many forms of targeted attacks and LAGFV based on shared extreme and overvalued beliefs, the intense and pathological preoccupation with an idea, person, or cause, otherwise referred to as pathological fixation, is an important variable to consider (Mullen et al., 2009; Meloy and Rahman, 2021). Fixation exists within a normal range of experiences, where many have a healthy everyday fixation related to their children, spouse, sports teams or other hobbies, which does not cause harm to themselves or others (Mullen et al., 2009). However, a gradual process leading to fixation can become pathological when it leads to social withdrawal and deterioration of normal functioning (Mullen et al., 2009; Meloy and Rahman, 2021). Fixation as a warning behaviour in threat assessment is distinguished by this increasingly dysfunctional preoccupation accompanied by negative affect, absolutist opinions, and harsh judgments about the object of the fixation, leading to a harmful impact on relationships and a decline in individual functioning (Reid Meloy et al., 2012). In severe cases, it can fuel a need or perceived obligation to commit a targeted attack (Mullen et al., 2009; Meloy and Gill, 2016; Meloy and Rahman, 2021; Brooks and Barry-Walsh, 2022; Ebbrecht, 2022). Building on a retrospective assessment of 377 targeted attacks, fixation was estimated to precede 81% of cases (Meloy and Rahman, 2021). As such, it is recognised as a proximal warning behaviour of lone-actor terrorism in the TRAP-18 (Meloy and Gill, 2016; Meloy, 2018).

Due to the significant role fixation appears to play in many cases of targeted attacks and violence, the Fixated Threat Assessment Centre (FTAC) was established in the United Kingdom and later in Queensland, Australia and New Zealand (James et al., 2014; Pathé et al., 2015; Meloy and Hoffmann, 2021; Wilson et al., 2021). FTAC is a joint task force of psychiatrists and law enforcement aimed at identifying and managing individuals at risk of committing violent attacks based on their intense preoccupation. While the FTAC model was initially introduced to manage the risk posed to the royal family and public figures, it was expanded in 2016 to account for fixated lone actors who posed an overall risk of committing acts of lone-actor grievance-fuelled violence (Logan et al., 2023). This expansion was implemented in light of the numerous terrorist attacks and mass violence cases perpetrated by fixated lone actors in recent years and the increased risk posed by individuals driven by a combination of grievances, ideological fixation, disturbed psychopathology and other vulnerabilities (Barry-Walsh et al., 2020; Wilson et al., 2021). Thus, based on the same conclusion, Rahman et al. (2021) presented three cognitive-affective drivers of fixation: *delusions*, *obsessions* and *extreme overvalued beliefs* (Rahman et al., 2020; Meloy and Rahman, 2021). Although the content, presentation, and subsequent behaviour of the three cognitive-affective drivers of fixation can appear similar at the surface level, the cognitive components, psychopathology, and aetiology are distinct (Sims, 1988; Meloy and Rahman, 2021).

Extreme overvalued beliefs (EOBs) were presented as a cognitive-affective driver for fixation, building on decades of research on overvalued ideas originally presented in the late nineteenth century by German psychiatrist Carl Wernicke and highlighted by European psychiatrists throughout the 20th century (McKenna, 1984; Sims, 1988; Jaspers, 1997; Veale, 2002; Rahman et al., 2019). Following the 9/11 terrorist attacks, overvalued ideas were reintroduced to threat assessment (McHugh, 2006). Overvalued ideas do not represent a disassociation from reality but exist as a solidary belief in line with pre-existing values and experiences and are thereby perceived by their holders as valid and justified (McKenna, 1984; Sims, 1988; Jaspers, 1997). In common speech, rather than being referred to as “*mad*” and out of touch with reality, individuals with overvalued ideas are frequently described as “*fanatics*” (McHugh, 2006; Freudenreich, 2020). EOBs are the only hypothesised driver of fixation based on an idea, belief, or ideology shared by a subculture. Their shared nature makes them distinct from other drivers of fixation, such as delusions and obsessions, which are both believed to be idiosyncratic, ego-dystonic and largely rooted in diagnosable mental illness. Delusions are false and unshared beliefs that remain fixed despite disconfirming evidence (Jaspers, 1997; Rahman et al., 2019; Meloy and Rahman, 2021). They often severely contradict the person’s education and cultural upbringing and are often accompanied by markers of psychosis (American Psychiatric Association, 2013; Andreasen, 2020). Similarly, obsessions are typically accompanied by egodystonic, persistent, intrusive and unwanted thoughts, images and/or urges, which are frequently anxiety-and shame-provoking (American Psychiatric Association, 2013, p. 826; World Health Organization, 2024, p. 696). They cause affected individuals significant distress, which leads many to perform mental or behavioural compulsions to neutralise their experiences, often associated also with Obsessive Compulsive Disorder (American Psychiatric Association, 2013; Meloy and Rahman, 2021).

Particularly in light of the current technological climate, EOBs as a driver for fixation have been used to capture and partly explain the growing risk of overvalued ideologies shared within subcultures in modern society, especially accounting for non-delusional and non-psychotic individuals driven to extreme acts of violence (McHugh, 2006; Rahman et al., 2016, 2019, 2020; Rahman, 2018; Meloy and Rahman, 2021; Mulay et al., 2023; Pierre, 2024). While mental disorders can and often do play a part in acts of violent extremism, psychopathology alone cannot explain extreme forms of violence. The Violence Project, an American non-profit association that examines mass shootings in the United States, has demonstrated the importance of this issue. One of their most significant findings is the critical limitation of the societal consensus that most mass shootings are solely due to severe mental illness (Peterson and Densley, 2021; Peterson et al., 2022). Besides, even when mental disorders play a role, the diagnoses of mental disorders within lone actor samples differ significantly. Schizophrenia, mood disorders, Autism spectrum disorder and personality disorders appear to be more prominent in these samples; however, their distribution and co-occurrence vary significantly, making the association and causality challenging to unravel (Corner et al., 2016; Gill et al., 2021).

Less researched, with much left to be explored, EOBs are of particular interest in light of the growing threat of domestic terrorism perpetrated by lone actors who adhere to beliefs that are shared and celebrated within subcommunities, especially online (Rahman et al., 2016, 2019, 2020; Rahman, 2018). However, by definition, EOBs growing more intense and emotional over time, being shared, ego-syntonic, and absolute, does not make them inherently violent or dangerous. Other belief systems that become an intensely important part of one’s identity, such as political or religious beliefs, can fall under the definition of EOBs. Even in other widely explored subcommunities where EOBs appear to grow rampant, such as Proud Boys, Sovereign Citizens and Involuntary Celibates, a vast majority of individuals do not become violent (Rahman et al., 2019). Despite being described as “… *a predominant motive behind global and homegrown violence and terrorist attacks*” (Rahman, 2018, pp. 1), on their own, EOBs do not provide sufficient explanation for violent attacks. Thereby, other crucial variables must be revisited in this context as their presence can increase the risk of fixated individuals becoming violent.

An aspect not wholly captured by the current definition of EOBs is the strong emotional commitment and transformation of a person’s identity which often arises from overvalued ideas (McKenna, 1984; Freudenreich, 2020). As we will argue below, such extreme changes to a person’s identity can drive pro-group violence, even if this involves great personal risk for the perpetrators. An important aspect of overvalued ideas, not present in delusions and obsessions, is the alignment with one’s personality, values and grievances, and their ego-syntonic nature leads to an increasingly growing emotional exacerbation and commitment (McKenna, 1984; Jaspers, 1997; Meloy and Rahman, 2021). The process of developing a fixation over time, growing more intense and emotional, as is the case for EOBs, can become central to the individual’s self-identity (Meloy and Rahman, 2021). A model presented by Veale (2002) argues that overvalued ideas are closely linked to idealised values, causing the overvalued ideas and idealised values to become a central part of the self and a growing part of one’s definition of the self (Veale, 2002).

A pathway of particular interest is when the fixation starts to alter or restructure one’s own identity and self and how that happens. When fixation becomes more overbearing and dangerous, and the social identity of the individual starts to change, it leads to another, more significant risk, which is referred to in threat assessment tools as *identification*. While acknowledging the importance of *identification,* we will argue in the following section that the construct may often be misapplied in the context of violence threat assessment and should be replaced with *identity fusion*. As mentioned above, previous studies conducted within a wide range of geographic and cultural contexts have shown that extreme forms of self-sacrificial violence are commonly linked to identity fusion, a powerful form of group alignment that is quite distinct from identification (Atran, 2016; Newson, 2019; Buhrmester et al., 2020). Most recently, quantitative analyses of the manifestos of convicted terrorists and online groups who engaged in offline violence found that perpetrators of violence were characterised by four statistically significant factors when compared to non-violent control groups: identity fusion, perceived outgroup existential threat, outgroup “othering” in the form of demonization or dehumanisation, and violence condoning ingroup norms (Ebner et al., 2022b, 2024; Ebner and Whitehouse, 2023). Connecting on these existing research streams, we will argue that EOBs are capable of leading to violent extremism when they produce identity fusion and contain elements of perceived outgroup threat, outgroup “othering,” and violence-condoning norms. Our extended framework will propose to integrate these additional factors into violence risk formulas used for psychological threat assessments.

### Identification versus identity fusion?

Both social and personal identities are uniquely important to our development and play a large role in how we see ourselves and the world. However, one’s identity can undergo deep and sometimes harmful transformations that can motivate extreme pro-group action, including acts of violence and terrorism. Two forms of group cohesion that have a significant impact on a person’s identity need to be distinguished: fusion or depersonalisation (Swann et al., 2012; Swann and Buhrmester, 2015; Whitehouse, 2018). Identity fusion entails a visceral oneness of self and group. In the process of identity fusion, the personal and group identities of an individual become functionally equivalent and are activated synergistically rather than hydraulically (Swann and Buhrmester, 2015). This means that the sense of personal agency of a fused individual is not diminished but strengthened. Depersonalisation, on the other hand, also referred to as identification, is a form of group alignment that leads to one’s personal identity becoming less central and apparent and the group’s identity becoming a ruling part of the individual existence (Swann and Buhrmester, 2015; Whitehouse, 2018).

At least two pathways to identity fusion have been distinguished: One pathway to fusion is through feelings of kinship based on perceptions of shared ancestry and phenotypic matching, and the other one is through shared transformative experiences. The latter pathway is associated with shared ordeals, such as warzone experiences, humiliating defeats or hazing procedures. Such experiences have been described as ‘imagistic’ because they create vivid “flashbulb” images that are stored in episodic memory, driving subsequent processes of reflection and interpretation (Whitehouse, 1995, 2000, 2004, 2021; Whitehouse and Lanman, 2014). When such experiences and processes of meaning-making are felt to be shared with others, this can lead to the fusion of personal and group identities (Whitehouse, 2021).

It is sometimes assumed that strong identification and identity fusion describe the same phenomenon, but this is not consistent with the evidence (Swann et al., 2009; Gómez et al., 2011; Swann and Buhrmester, 2015). Unlike the imagistic process that leads to fusion based on the sharing of personally transformative experiences with other members of the group, identification is thought to result from the sharing of socially learned group identity markers (e.g., group-defining beliefs and practices) that are not anchored in personal autobiographical memories. Since identification is based on the sharing of schemas and scripts stored in ‘semantic’ memory (Whitehouse, 2000, 2004) rather than in personal experiences stored in ‘episodic’ memory, activation of group identities is depersonalising, making personal identities less accessible (Whitehouse and Lanman, 2014). The latter process is typical of doctrinal religions, where the unification of adherents is rooted in shared beliefs and practices rather than in shared imagistic experiences (Whitehouse, 2021).

The differences between the ‘imagistic mode of religiosity’ and the ‘doctrinal mode of religiosity’ may shed light on the fixation process leading to identity fusion or identification. A key element of the ‘imagistic’ pathway is ‘spontaneous exegetical reflection’ (Whitehouse, 2004) – the process of reflection on the meaning of personally impactful and transformative experiences that become essential to the autobiographical self (Whitehouse and Lanman, 2014). To the extent that reflecting deeply on a life-changing experience can lead to a permanent change to the autobiographical self, sharing such experiences with others can serve to fuse one’s personal and group identities together. Several empirical studies support this model. For example, surveys and priming studies suggest that reflecting on traumatic experiences of The Troubles in Northern Ireland creates fusion with a community of fellow sufferers, whether on the nationalist or unionist side of the conflict (Jong et al., 2015).

Moreover, such experiences and processes of spontaneous exegetical reflection can drive fusion even when they are shared vicariously rather than directly. For example, when the story of the shooting of Cecil the Lion went viral in 2016 (Whitehouse, 2024b), many people around the world reflected on the episode over time and – to the extent that they felt that this process was shared by lion conservationists – experienced fusion with them as a result (Buhrmester et al., 2018). When fusion is based on vicariously shared experiences and reflection in this way, it can also lead to identification in ways that are typical of the doctrinal mode of religiosity (Whitehouse, 2021). That is, people are well aware that they were not present at the killing of Cecil the lion and that the very possibility of sharing the experience variously with supporters of lion conservation is based on shared values and doctrines rather than undergoing the same episodic event together. In such cases, fusion is therefore extended to a generic group category with which one identifies. Similar processes may occur when, for example, religious groups become extendedly fused as a result of vicariously shared experiences of outrage at events reported in the media (Kavanagh et al., 2020). Whereas local fusion occurs in the context of relational ties that members of relatively small homogenous groups may develop based on directly shared experiences, such as warzone battles that are fought together, extended fusion can occur when individuals “project relational ties onto relatively large collectives composed of many individuals with whom they may have no personal relationships” (Swann et al., 2012).

**Figure 1:**
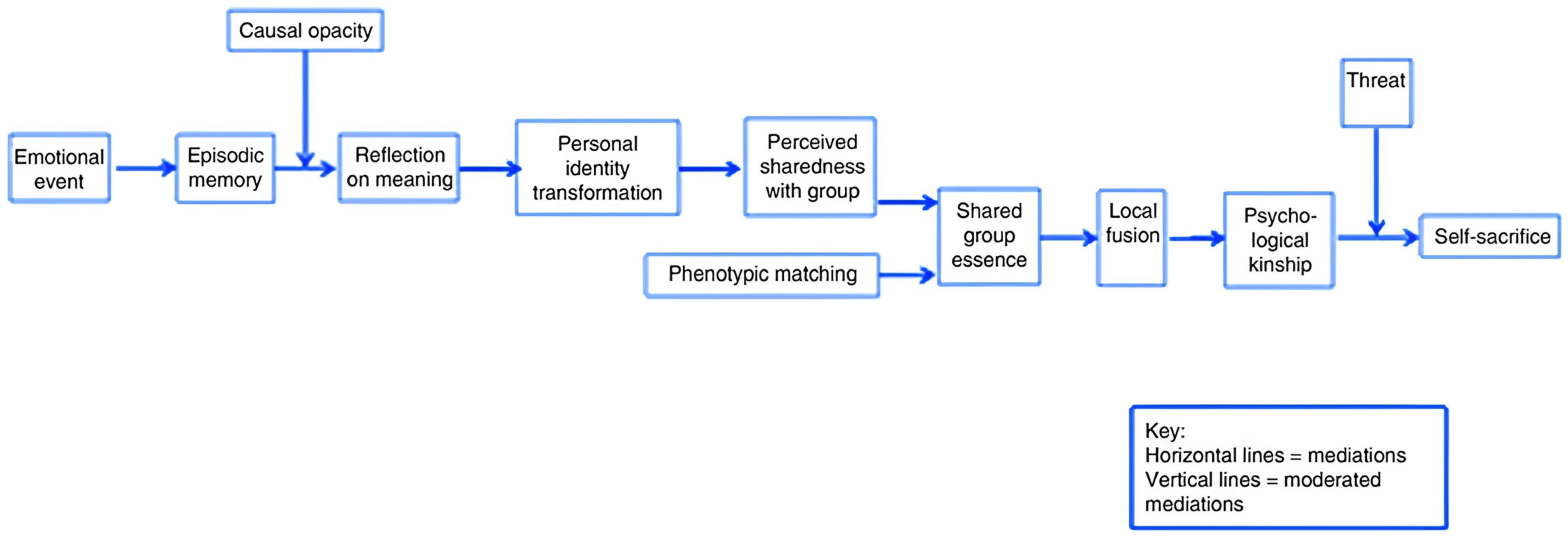
Pathways to Fusion and Self-sacrifice reproduced from Whitehouse (2018, p. 2) reprinted with permission.

Identity fusion, *per se,* is not necessarily associated with negative or harmful outcomes. For example, most people are fused with their families and would go as far as to put their own lives on the line for their relatives. Likewise, soldiers in warzones often show high levels of fusion and are more willing to sacrifice their lives for one another, which is – at least from a military perspective – perceived as desirable. In 2018, the fusion-plus-threat model (see Figure 1) proposed that identity fusion tends to have significant effects on individual escalation to self-sacrificial violence when it is combined with a perceived existential threat to the ingroup (Whitehouse, 2018). In response to the presentation of this model, a range of additional factors were suggested in published peer commentaries. We incorporated these factors into a systematic analysis of the communication materials of perpetrators of terrorism and violent extremism. Our findings offered further support for the theoretically grounded fusion-plus-threat model. Indeed, our recent studies confirmed that the psychological formula for extreme violence might include a combination of identity fusion, existential threat perception, hostility toward an outgroup and violence condoning ingroup norms (Ebner and Whitehouse, 2023; Ebner et al., 2022a, 2022b; 2024).

While local fusion has been observed in the context of pro-group violence carried out by terrorist groups, militia movements or battalions (e.g., Whitehouse et al., 2014), extended fusion is likely to be the more relevant form in the context of violent extremism, as most perpetrators in this category will not have previously developed personal relationships with other ingroup members but tend to be fused with wider collectives such as white Europeans, the U.S. al-right or the QAnon community. Notably, this calls into question the individualistic approach dominant in threat assessment protocols, which largely overlooks identity fusion and group dynamics that can and do play a significant role in violent extremism (Gill et al., 2014; Ebner et al., 2022a). Since fusion can extend to a wider ingroup and does not necessarily rely on direct contact or relational ties, it can also be a significant contributor to lone actor violence. As fused members tend to perceive other ingroup members as family, kinship language and metaphors of shared blood may serve as linguistic proxies for identity fusion (Whitehouse and Lanman, 2014). For example, individuals who are fused with a wider ingroup of a collective based on race might call their peers “my white European brothers and sisters” or speak about “white blood” (Ebner et al., 2022a).

Despite this, identification continues to be accorded a central role in threat assessment tools, where it refers to the spiral from fixating on a specific subject to considering oneself a warrior or agent for a belief system or ideology or identifying with a particular offender with the same set of beliefs (Meloy et al., 2021a). It is often associated with pseudo-commando behaviour and wanting to fight for a certain belief or ideology (Meloy et al., 2015b) and may also be coded when an offender stockpiles or collects weapons and has close association with military equipment (Meloy et al., 2021a; Brugh et al., 2023). It has been widely covered in forensic evaluations of lone-actor terrorists and offenders (Meloy and Gill, 2016; Meloy, 2018), perhaps understandably, as the term “lone-actor” implies that group dynamics do not play a role. We argue however, that this should be reconsidered. Our goal is to explore the possibility that while an attack may be perpetrated by a lone actor, both shared beliefs (in the form of EOBs) and shared identities (in the form of identity fusion) play a key role in the pathway to violent extremism.

To complicate matters further, the term *identification* in threat assessment and across disciplines appears to be used in a manner that contradicts the evidence from social psychology and evolutionary anthropology about its causes, mechanisms and behavioural outcomes. For example, in the recently published book Violent Extremism (Logan et al., 2023), a description of the 3 N model and identification reads, “group processes and networks may evoke strong group dynamics, which enable individual and group identities to *fuse* and thereby distort acceptable norms. These network mechanisms facilitate engagement and strong identification with like-minded individuals, which reinforces support for political violence” (Gill and Rottweiler, 2023, p. 22). While this description is consistent with identity fusion, the process is attributed to “identification” in forensic psychology, which in evolutionary anthropology and social psychology refers to the depersonalisation of the individuals rather than the fusing of group and individual identities (Swann and Buhrmester, 2015; Whitehouse, 2018).

The conflation of terminology around forms of group alignment has resulted in identification being frequently cited as a key proximal warning indicator, even though fusion likely plays the more significant role in pathways to violent extremism. Fusion has almost entirely been neglected from current risk assessment protocols despite the theoretical underpinnings, as well as the differences between identification and fusion, being essential for the creation of a more robust threat assessment of lone actors. As briefly mentioned above, the focus on individual vulnerabilities in assessing the risk of LAGFV has come at the expense of theoretical grounding based on insights from other disciplines, such as group psychology, which may offer more holistic approaches (Meloy, 2018; Meloy et al., 2021b; Gill and Rottweiler, 2023). One of the factors that may contribute to confusion is that extended fusion to large-scale groups is often highly correlated with strong forms of identification. In such cases, beliefs, practices, and symbols serving as essential emblems for the extended group may come to be seen as inviolable and non-negotiable ‘sacred values’ in Atran’s sense (Whitehouse, 2024a). The role of idealised values in the process of overvalued ideas becoming central to one’s identity, previously highlighted in psychiatry and psychology (Veale, 2002), shares clear similarities with the role of sacred values in research on belief formation and identity fusion (Atran and Axelrod, 2008; Whitehouse, 2024a). Both constructs may play a central role in driving changes to one’s identity and perception of the self.

High levels of group identification can lead to commitment to extreme ideologies, yet this form of group cohesion is not thought to be sufficient to drive extreme forms of pro-group action such as violence and terrorism (Swann et al., 2012; Swann and Buhrmester, 2015; Whitehouse, 2018). Studies have shown that the self-preservation instinct tends to trump group loyalty when individuals with high levels of group identification face decisions of life and death. This was further supported by statistical terrorist manifesto analysis, which showed that identification levels were high across both violent and non-violent extremist authors (Ebner et al., 2024). Highly fused individuals, however, are more risk-taking when pro-group action might imperil survival. As threats to the group are taken personally, they might even risk life and limb for the group, for example, by throwing themselves on a grenade or by carrying out a suicide bombing. A growing body of research from different geographical, cultural and religious contexts has provided evidence for this fusion-violence link (Whitehouse et al., 2014; Atran, 2016; Newson, 2019; Buhrmester et al., 2020; Kavanagh et al., 2020).

With the link between identity fusion and extreme self-sacrifice in mind (Whitehouse, 2018), we revisit the study of violent extremism. The terminology and primary focus of violent extremism have caused ongoing debate, but it has been defined as a belief that violence against a perceived outgroup is an unavoidable component for the survival or success of the ingroup (Berger, 2018). Specifically, for the current review, we focus on an overarching behavioural component of violent extremism instead of the often-assumed ideological commitment to violence. That is extreme self-sacrifice and the willingness to engage in high-risk activities, including terrorism, on behalf of an ingroup (Ebner et al., 2024), which, as previously stated, can build on local fusion in the context of relational ties but can also build on extended fusion with a wider perceived ingroup that does not rely on direct contact. While group dynamics and self-sacrificial dimension in violent extremism have been neglected in research on lone actors, the shared and overlapping psychological elements tend to be overshadowed by a strong focus on ideological components. However, using the behavioural outcome as a guiding light, instead of ideological elements, we factor in the emerging terrorist and extremist threats highlighted in recent reports and the media. The preoccupation with ideology as the main motivator of extreme violence has recently been challenged, and the need to adapt to observable patterns must be addressed to account for at-risk individuals who do not fall into previously defined ideological categories (Gartenstein-Ross et al., 2023; Bush, 2025). Recent lone-actor attacks show that perpetrators do not necessarily fixate on a specific ideology but often appear to be “*fixated on extreme violence, seemingly for its own sake*” (Starmer, 2025). As the absence or incoherence of ideological motivations underpinning violent extremism is increasingly recognised, it becomes easier to appreciate the need to move the focus onto behavioural components that characterise many forms of extreme violence, including terrorism and lone-actor grievance-fuelled violence.

### EOBs and identity fusion: an underexplored relationship

Our framework incorporates the literature on both EOBs and fusion, arguing that there is an underexplored relationship between these two violence risk factors. Importantly, fixation may stem from personally transformative traumatic events in much the same way as military hazing rituals and tribal initiations (Whitehouse, 2021). Participants may be quite convinced that their experiences of suffering and meaning-making are not only personally transformative but also group-defining, thus leading to the fusion of personal and group identities.

**Figure 2:**
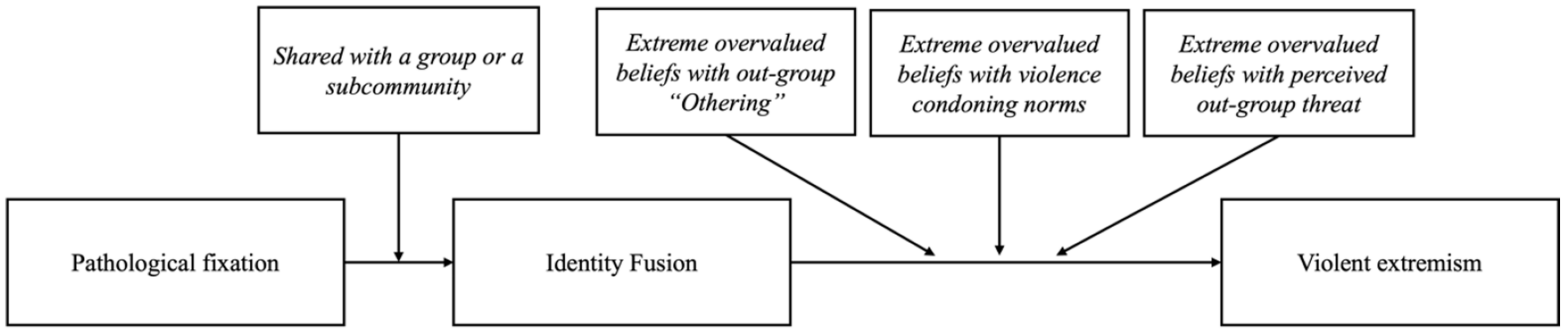
Hypothetical EOB-plus-fusion pathway to violent extremism.

Based on existing theory, we would not expect either EOBs on its own or fusion on its own to adequately explain why individuals engage in acts of extreme violence. However, when fixation on shared subcultural ideologies (EOBs) is capable of generating identity fusion and when it encompasses shared beliefs of an existential threat to the ingroup, outgroup ‘othering’ and violence-condoning norms, we would expect this to become a powerful driver of extreme progroup violence. This might even be the case with an extended ingroup, rather than only for an immediate social network. As this pathway would apply to multiple forms of self-sacrificial violence, we use the overarching concept of ‘violent extremism’ in our framework, which encompasses acts of lone-actor grievance-fuelled violence. Instead of solely focusing on ideological violence or the ideological component often highlighted in violent extremism (for example, White Supremacy), we refer to self-sacrificial violence on behalf of the ingroup (for example, violence to protect the wider ingroup of White Europeans) (Ebner et al., 2024). Figure 2 displays this hypothesised pathway. We have made a distinction between the process itself (EOBs, fusion and violent extremism) and the essential components for the process to develop, which includes the sharing of belief or ideology which underlies the fixation and the specific ingredients of EOBs that have been found to significantly increase risk of violence.

To further explore the EOBI framework proposed, we will review three cases of lone-actor grievance-fuelled violence. There, the elements proposed in Figure 2 are analysed specifically for each case. In Table 1, we highlight these components for each perpetrator, and in the following section, we give a detailed analysis of each case study. While explaining this previously unexplored pathway, we will also recognise and describe other psychological, psychiatric, and social contributors that can increase individual vulnerabilities to fixation, fusion and extreme violence.

**Table 1:** The EOBI framework applied to the three case studies.

	Anders Breivik	Dylann Roof	Brenton Tarrant
Fixation	AB became fixated on absolute and binary anti-immigration and anti-Islam beliefs, which he shared within the wider counter-jihadist far-right, such as the idea that “*Islam is taking over Europe*”.	DR held strong, binary and emotional beliefs about race, for example that white people are being attacked and killed by black people. His beliefs developed online through white supremacist websites and forums and grew over time.	BT became fixated on jihadist terrorism and believed there was a “great replacement” of white people, an absolute and binary belief he shared with a wider community of Reddit and 4Chan.
Shared with a group or a subcommunity	His beliefs were shared with White Supremacist, right-wing extremists and Neo-Nazi groups.	His beliefs were shared with White Supremacist, right-wing extremists and Neo-Nazi groups.	His beliefs were shared with a group of White nationalists on 8Chan.
Identity Fusion	AB’s identity was fused with white Europeans who he believed were under attack by Muslim immigrants, frequently referring to his ingroup as “brothers and sisters”.	DR’s identity was fused with a wider ingroup of white Americans. He used metaphors of shared blood with his ingroup and wrote about “good white blood” being under attack.	BT referred to white people as his “brothers” and committed the attack on behalf of his metaphorical family and his people to secure a future for white children.
Extreme overvalued beliefs with outgroup “Othering”	AB referred to Islam, Cultural Marxists, and multiculturalist elites as “the enemy” and “traitors” and saying they will be punished for their crimes.	DR used degrading and racist language, such as “animals”. He stated that Black people had lower intelligence and impulse control.	BT referred to Muslim people as enemies and spoke of Europeans who had lost their lives foreign invaders.
Extreme overvalued beliefs with violence condoning norms	AB frequently justifies his violent view and calls for a civil war to prevent the Islamisation of Europe. He promoted and favoured extreme violence to reach his goals.	DR viewed violence as the only option, feeling obligated to act in light of an impending race war.	BT had a strong endorsement and glorification of violence and called for violence against many people, including ‘high-profile’ enemies.
Extreme overvalued beliefs with perceived outgroup threat	AB focused on perceived threats of migration and multiculturalism and the ethnic cleansing in Europe, outlining the need to save Western Europeans from rapes, beating, robbing and murders.	DR had apocalyptic beliefs that white people were being systematically killed and attacked by ethnic minorities and believed there was going to be a race war.	BT believed there was a great replacement of white people and an ongoing white genocide with ethnic, cultural and racial eradication of white people of European descent by non-white migrants.
Violent extremism	Lone-actor terrorist attack in Oslo and Utøya targeting the Norwegian Labour Party	Mass shooting at the Emanuel African Methodist Episcopal Church in Charleston	Targeted attack on two Mosques in Christchurch New Zealand

### Case studies

In the following section, we will examine three case studies to further explore the relevance of our EOBI framework by assessing convicted perpetrators of lone-actor grievance-fuelled violence. The case studies will focus on three high-profile LAGFV acts carried out by Anders Breivik in Norway in 2011, Dylann Roof in the United States in 2015 and Brenton Tarrant in New Zealand in 2019. While acknowledging that all three offenders were fixated on white nationalist ideology and based in Western cultures, these cases were selected as they have previously been identified in research on extreme overvalued beliefs using the model of the path to intended violence (Faccini and Allely, 2016; Allely and Faccini, 2019; Allely et al., 2023) and the TRAP-18 (Kupper and Meloy, 2021), as well as studies on identity fusion (Ebner et al., 2022a, 2024). Thereby, the goal was to build on previous research and draw on independent assessments of these cases, while maintaining as much geographic diversity as possible (Norway, USA and New Zealand) to avoid focusing on cases from a single country or continent.

Our case study analysis will focus on factors identified as important in previous assessments: the fixation being shared, simple/binary and ego-syntonic, associated with high affective intensity and grievance, and an abnormal personality but no psychotic symptoms – all of which are outlined in previous studies to explore EOBs (Rahman et al., 2016; Meloy and Rahman, 2021). We will outline the backstory and pathway in each case, especially illustrating the role of EOBs as a driver for pathological fixation and the role of identity fusion. In line with our proposed framework, we will also include an assessment of specific EOB ingredients, including violence-condoning norms, ‘othering’ of the outgroup, and perceived outgroup threat. The analysis will be based on primary materials from the offenders themselves (their manifestos), psychiatric and psychological evaluations prior to and following the offences, biographical accounts, competency reports, and other case studies published following the attack.

#### Anders Behring Breivik

On the 22nd of July in 2011, after years of planning, 32-year-old Anders Behring Breivik committed the worst terrorist attack in the history of Scandinavia, taking the lives of 77 individuals and wounding over 240 others (Borchgrevink, 2013; Virtanen, 2013; Faccini and Allely, 2016). The attack began with a bomb detonating in the centre of Oslo, close to the Prime Ministry, resulting in city-wide panic, and all police officers immediately dispatched to the scene. Then, Breivik, dressed as a police officer himself, travelled to Utøya, where a yearly youth summer camp was being hosted on behalf of the Norwegian Labour Party. Upon his arrival on the island, Breivik began shooting indiscriminately at crowds of mostly young people. Eight individuals lost their lives in the explosion in Oslo and a further 69 at Utøya, with a total of 59 victims under the age of 21 (Borchgrevink, 2013; Melle, 2013; Ranstorp, 2013; Richards, 2014). Prior to his attacks, Breivik posted his own manifesto online, titled “*2083 – A European Declaration of Independence*” (Breivik, 2011; Johnsen, 2014). This 1,500-page manifesto included an outline of his reasoning, motivation and ideology and an interview with “The Knights Templar.” However, it was soon discovered that he acted alone, the Knights Templar was, in fact, himself and large parts of his texts were copied from other right-wing sources (Kundnani, 2012).

Breivik had a history of difficulties. While he recounts his youth as happy, healthy, and socially thriving, this significantly contradicts accounts from friends and family about his childhood. He had a dysfunctional relationship with his mother, and his father was hardly present in his life. At age four, his mother sought the help of a psychiatrist to manage his behaviour, which she described as aggressive and demanding (Melle, 2013; Gullestad, 2017). Breivik did have some friends growing up and recounts being good at socialising (Breivik, 2011), but his friendships and later romantic encounters all dissolved at some point. He explains this in numerous ways, including that he wanted to focus on his education, goals or, eventually, his mission (von Brömssen, 2013; Gullestad, 2017). He suffered through a lot of rejection in his life from his absent father and rejection from gaming communities, political groups and some romantic interests. He declared bankruptcy five years prior to his attack, resulting in him having to move back in with his mother. This is recounted as a major turning point, where he likely developed a gaming addiction (Pantucci, 2011; Melle, 2013; Meloy et al., 2015a).

During his trial, Breivik was originally diagnosed with schizophrenia, and his fixation was attributed to grandiose delusions, making him eligible to plea for the insanity defence and diminished responsibility. However, this was widely debated, which resulted in a re-evaluation by a second group of forensic psychiatrists who questioned the original conclusion that he was psychotic. The re-evaluation found that he did not have a psychiatric disorder or delusion at the time of his offence (Wessely, 2012; Melle, 2013; Rahman et al., 2016). Rather, they believed he was motivated by an extreme right-wing political ideology and identified as a warrior for a cause that was shared by other members of white supremacist groups. Breivik was diagnosed with narcissistic personality disorder with pathological lying (pseudologia fantastica) (Melle, 2013). This was accompanied by other malignant personality traits, a lack of empathy and some evidence of traits associated with Autism spectrum disorder (Faccini and Allely, 2016). Despite some disagreement, it was determined that the second evaluation had more merit, and it was concluded that he had EOBs heightened by his self-aggrandisement rooted in narcissistic personality disorder (Melle, 2013; Rahman et al., 2016).

Breivik had an intense emotional commitment to his beliefs, and his attack took years of planning, both strategically and financially. His fixation was a primary driver in his life, and most of his time, finances and focus were devoted entirely to his ideas and mission (Pantucci, 2011; Melle, 2013; Meloy et al., 2015a). His beliefs were shared with white supremacist groups, right-wing extremists and Neo-Nazis and based on the same perceived threats of migration and multiculturalism (Wessely, 2012; Melle, 2013; Rahman et al., 2016). It is explicit in his writing and subsequent publication that Breivik’s fixation grew over time, and his attacks took years of planning (Breivik, 2011; Melle, 2013; Rahman et al., 2016). Drawing on our previous psycholinguistic analysis of terrorist manifestos, we argue that his EOBs were characterised by the three specific ingredients outgroup ‘othering’, perceived outgroup threat and violence condoning norms.

- Outgroup ‘othering’: Breivik (2011) frequently discusses Islam, Cultural Marxists, and multiculturalist elites, referring to them, for example, as “the enemy” (p. 18) and as “traitors” (p. 797) and saying they will be punished for their crimes (p. 1131). He draws clear distinctions between white Europeans and other races and religions.
- Perceived outgroup threat: Breivik recounts that he was saving Europe as he believed there to be a severe threat stemming from Muslim immigrants and the ever-threatening plans of the ethnic cleansing of white Europeans (Melle, 2013). There are many accounts of his perceived threat of the outgroup in his manifesto, which were non-European immigrants (especially Muslims). For instance, the manifesto says: “*I truly fear for the future of Europe. How can I procreate knowing that we are heading for cultural suicide*?” (p. 1359) and “*the Nordic tribes will become extinct if we do not resist and seize political and military control of our countries*” (p. 1156). He lists out expected rapes, beating, robbing and murders of Western Europeans by Muslim immigrants to outline the urgency to respond to the threat of “*the regime*” (p. 1030).
- Violence-condoning norms: Throughout his manifesto, Breivik references other violent white supremacist groups such as the Ku Klux Klan and skinheads. He frequently justifies his violent view and calls for a civil war to prevent the Islamisation of Europe (Breivik, 2011). He writes that his followers need to “embrace and familiarize themselves with the concept of killing women, even very attractive women” (p. 941). His increased preoccupation began to promote and favour the use of violence to reach his goals (Meloy et al., 2015a).

Breivik displayed clear signs of extended identity fusion, in particular a perceived oneness of his personal identity with the wider ingroup of white Christian Europeans as well as with the narrower group of the Knights Templar. As outlined in previous natural language processing (NLP) analysis (Ebner et al., 2022a, 2024), his elevated identity fusion levels were marked through statements such as “*How many of our sisters have and will be raped by Muslims*?” and “*How many of our brothers and sisters will commit suicide due to these atrocities*?” (Breivik, 2011, p. 1031). The terms he used in the parts of the manifesto that he wrote himself refer to a sense of biological kinship with the ingroup, which is a strong indicator of fusion and frequently observed in self-sacrificial texts of perpetrators of violence (Whitehouse, 2018; Ebner and Whitehouse, 2023). In line with the fusion-plus-threat model (Whitehouse, 2018), the identity fusion proxies tend to co-occur with a perceived threat to the ingroup. Looking at the origins of fusion, there is also evidence of personally transformative experiences that Breivik shared with his ingroup. Notably, analyses of his manifesto, psychiatric assessments and subsequent case studies showed that Breivik displayed a combination of personal grievances (e.g., his own bankruptcy and experiences of rejection) and political grievances (e.g., his belief that “Cultural Marxists” were destroying white European societies) (Breivik, 2011; Melle, 2013; Faccini and Allely, 2016). In his manifesto, he described having been a victim of eight assaults and knowing more than 20 people who had been beaten or robbed, as well as two women who had been raped by Muslims (Breivik, 2011, p. 1390). As outlined earlier in this paper, identity fusion tends to be caused by the sharing of dysphoric experiences with one’s ingroup (Jong et al., 2015).

#### Dylann Storm Roof

In 2015, 21-year-old Dylann Roof walked into the Emanuel African Methodist Episcopal Church in Charleston, South Carolina and joined a prayer session. Following the session, he proceeded to pull a gun he had brought with him and shoot ten people, killing nine (Bass, 2021; Sizoo et al., 2022). Prior to his attack, he published his manifesto titled “The Last Rhodesian” (Roof, 2015). In his 5-page manifesto, he addressed his beliefs and ideology in detail, including the conclusion that he felt obligated to act, instigating a civil war and stopping the attack on the white race (Allely and Faccini, 2019; Bass, 2021). A pivotal moment in Roof’s life, in regard to his beliefs and ideology, was the murder of Trayvon Martin—a black, unarmed teenager who was killed by police officer George Zimmerman. Following the news of that case and what he viewed as unfair coverage, Roof began researching and reading about “black-on-white” crime (Payne, 2015; Bass, 2021), noting that he had “*never been the same since that day*” (Roof, 2015, p. 1). In 2013, two years prior to his attacks, Roof began reading extensively online about racial violence against white people, notably googling topics on black-on-white crime, “Muslim-gang rape” and “Jewish control” (Sizoo et al., 2022).

Roof had a number of difficulties growing up. His parents divorced when he was very young, and he appeared to struggle with social relationships throughout his life. He lost his best friend in an accident and had little social interaction after that. Overall, he had great difficulty making friendships, did poorly in school and could not maintain employment (Loftin, 2016). He developed severe anxiety, became isolated and later developed an addiction to alcohol and marijuana (Payne, 2015). He was withdrawn and isolated and spent unlimited time on websites that fuelled his fixation (Loftin, 2016; Maddox, 2016). Through psychological- (Loftin, 2016), neuropsychological- (Moberg, 2016) and psychiatric evaluations (Maddox, 2016), it was determined that Roof had Autism spectrum disorder, which caused a number of difficulties and was a formative factor in his pathway to violence. He was extremely rigid and had difficulty shifting cognitive tasks and mindset (Loftin, 2016). While Roof displayed signs of grandiosity at times (Maddox, 2016), he appeared to be extremely concerned with his reputation following his attack, cared considerably about the opinions of other people, and feared being judged and criticised (Ballenger, 2017; Allely and Faccini, 2019). His family noted that he was passive, socially awkward, anxious and a bit bizarre (Moberg, 2016).

It is relatively common with Autism spectrum disorder to show signs of comorbid psychiatric conditions, and Roof was diagnosed with Specified Schizophrenia Spectrum and Other Psychotic Disorders during his psychiatric evaluation (Maddox, 2016; Moberg, 2016). It was believed that the psychiatric conditions were developing alongside his shared racist beliefs rooted in white supremacist ideology, along with notable signs of transient ideation marked with paranoia (Maddox, 2016) and grandiose and magical thinking (Moberg, 2016). Despite his Autism spectrum disorder being associated with psychotic spectrum features, there was a lack of acute psychosis or mood disturbance (Moberg, 2016). His diagnosis was debated and later disregarded in a secondary competency evaluation, where the expert found no sign of psychosis or incompetency but rather that he had logical (albeit false) explanations for his behaviour and beliefs (Ballenger, 2017). His justification for his racial beliefs and actions had been consistent since long before his attacks, which underlies the fact that the crime was “*not a product of disease or defect*” but a result of his beliefs and prejudice, which grew over time (Ballenger, 2017)^2^. Thereby, Roof’s fixation has been attributed to EOBs rather than delusions or obsessions (Rahman et al., 2021).

Analyses showed that Roof’s fixation developed mainly through the Internet (Norris, 2017). He stated in his psychiatric evaluations that he had uncomfortable feelings, which he found he could explain based on his own research on race and crime, leading to his beliefs becoming increasingly “*fixed and unchecked by reality*” (Maddox, 2016, p. 5). His fixation was associated with strong emotion and a sense of urgency, noted in his manifesto and subsequent psychological- (Loftin, 2016), neuropsychological- (Moberg, 2016) and psychiatric evaluations (Maddox, 2016). In his manifesto, he quoted American History X: “*I see all this stuff going on, and I do not see anyone doing anything about it. And it pisses me off*” (Roof, 2015, p. 5). While his fixation certainly developed through his online searches, he was known for his racist views. His views were shared by his acquaintances at school and remained unchecked (Loftin, 2016). He stated that, upon conducting his search online, everything started to make sense “like an epiphany” (Moberg, 2016). Roof’s beliefs developed online, where he found white supremacist websites that shared his views, slowly radicalised him and convinced him of the urgency and need to plan a violent attack himself (Loftin, 2016; Maddox, 2016; Sizoo et al., 2022). Roof’s fixation became more refined, absolute and overbearing over time. While it was in line with his some pre-existing racist views, when he consumed content almost exclusively on racist blogs and alt-right websites, it caused his shared fixation to grow and become all-encompassing (Allely and Faccini, 2019). Like in Breivik’s case, we found elements of outgroup ‘othering’, perceived outgroup threat and violence condoning norms in his beliefs.

- Outgroup ‘othering’: In his manifesto, Roof stated that black people have lower intelligence and impulse control but higher testosterone, which leads to violent and aggressive behaviour according to him. He called them “lower beings” and “brute animals” (p. 2). He noted, “*Anyone who thinks that White and black people look as different as we do on the outside, but are somehow magically the same on the inside, is delusional. How could our faces, skin, hair, and body structure all be different, but our brains be exactly the same?*” (Roof, 2015).
- Perceived outgroup threat: In both his manifesto and subsequent psychiatric evaluations Roof said that he believed that white people were under attack by ethnic minorities and, most of all, by black people (Roof, 2015; Maddox, 2016). He was convinced that “*blacks are taking over the world*” and that no group, like the Ku Klux Klan or Skinheads, were doing anything other than complaining on the internet (Roof, 2015, p. 5). Roof was severely influenced by what he read about brutal murders of white people when googling black-on-white crime, which was mostly based on misinformation and propaganda published by white supremacist websites. He was preoccupied with the idea that white people were under attack, that “*thousands of white women were being raped each year by black men*” (Moberg, 2016), and that eventually white people would become extinct and there would be a race war (Maddox, 2016).
- Violence-condoning norms: Roof felt justified (even obligated) to pursue his attack and was certain that violence was the only option in light of his apocalyptic beliefs that white people were being systematically killed and attacked (Allely and Faccini, 2019; Bass, 2021). Roof was driven, at least in part, by his perceived need to start a race war (Norris, 2017).

Identity fusion was observed through NLP analysis of Roof’s manifesto (Ebner et al., 2022a, 2024). He used phrases such as “good white blood worth saving” (Roof, 2015, p. 4), which relates to metaphors of shared blood as a linguistic proxy of fusion and a common pattern in violent self-sacrificial texts (Ebner et al., 2022a). Similar to Breivik’s identity fusion with white Europeans, Roof’s personal identity started to fuse with his wider racial ingroup when he reflected on his shared dysphoric experiences. Roof thought more about his personal racial grievances and perceived victimhood when he started reading about racial crimes against other white people on white supremacist websites. Upon conducting his own “research,” he experienced strong negative feelings associated with helplessness, depression and feelings of low personal significance and self-esteem. He believed news of black-on-white crime was not getting any coverage (Allely and Faccini, 2019).

#### Brenton Harrison Tarrant

The most recent example covered in this review is the Christchurch Mosque Attack in New Zealand. In 2019, a 28-year-old man named Brenton Tarrant attacked two mosques in a span of 37 min, murdering 51 individuals and injuring a further 49 (Macklin, 2019; Baele et al., 2023). The massacre was filmed using a camera he attached to his helmet, using Facebook Live, where he had shared a link online to white supremacist forums on Reddit, 4Chan, 8Chan, and Twitter, encouraging his fellow users to tune in (Macklin, 2019; Baele et al., 2023). The day before his attacks, he published his own manifesto to outline and describe his beliefs and motives, titled “The Great Replacement” (Tarrant, 2019; Obaidi et al., 2022). In his 74-page manifesto, he wrote about his goal of protecting the white race from Muslim immigrants. He was consumed by alt-right and white supremacist agendas and had even decorated his guns and machinery with “Mein Kampf” and other neo-Nazi symbols, as well as the names of previous far-right attackers (Hendrix and Miller, 2019; Calhoun and Weston, 2021; Baele et al., 2023).

Tarrant grew up in New South Wales; he described himself as “*just an ordinary White man, 28 years old. Born in Australia to a working-class, low-income family*” (Tarrant, 2019, p. 5). He worked as a fitness instructor, offered free classes to children and had no criminal history (Chung and Laura, 2019; Macklin, 2019). He was extremely introverted and had social anxiety from a young age (Royal Commission of Inquiry, 2020). Prior to his attack (and through much of his life), Tarrant was isolated and had no close friends, the main connections in his life were his mother and sister (Royal Commission of Inquiry, 2020; Allely et al., 2023). His mother noted that his personality changed significantly after his parents’ separation and he became even more withdrawn and isolated, which appears to have developed through adulthood (Royal Commission of Inquiry, 2020). He displayed signs associated with autism and clear sociopathic traits (Allely et al., 2023). A combination of this was demonstrated in his extreme lack of empathy for the “outgroup” (Royal Commission of Inquiry, 2020). His fixation grew over time and was shared, relished and celebrated but was not directly attributed to a severe mental disorder or detachment from reality (Allely et al., 2023).

Research indicates that he became fixated on jihadist extremism and terrorism at least two years prior to his attack (Counter Extremism Project, 2023). A defining moment for Tarrant was an Islamist terrorist attack in Sweden in 2017, where a truck drove into a group of people, killing four, including a young girl named Ebba Åkerlund. This changed the trajectory of Tarrant’s life: only six months later, he applied for a gun permit (Pratt, 2020) and took two years to plan his attack (Tarrant, 2019, p. 16). He was heavily influenced by Anders Breivik, stating that he received Breivik’s blessing for his mission (p. 12). He also names American politician Candace Owens as an important influential figure but states that, even for him, some of her views were too extreme (Tarrant, 2019, p. 17).

Tarrant’s rage and extreme views were directed towards Muslims (Allely et al., 2023). It is observed in Tarrant’s manifesto that he was emotional about his cause, with statements such as “… *my despair turned to shame, my shame to guilt, my guilt to anger and my anger to rage*” (Tarrant, 2019, p. 9). Tarrant had shown racist behaviour for a long time, ever since he was a child (Royal Commission of Inquiry, 2020). His evolving hatred towards outgroups aligned closely with his pre-existing values, culture and beliefs, which led him to seek more information from white supremacist forums and websites and donate to far-right extremist organisations such as Generation Identity (Allely et al., 2023). His beliefs were mainly in line with white nationalism, with an added murky combination of anti-Muslim ideologies, Christian extremism, eco-fascism, siege culture and accelerationism, aligning closely with the aforementioned “salad bar extremism” (Am and Weimann, 2020; The Soufan Center, 2021; Counter Extremism Project, 2023; Meier, 2023).

Tarrant’s pathway to violence could accurately be described as “going down a rabbit hole,” where the internet played a definitive and formative role in his later attack (Allely et al., 2023). He specifically discusses YouTube, 4Chan and previous far-right attackers, such as Anders Breivik and Dylann Roof, as formative and influential for his development (Am and Weimann, 2020). His fixation grew over two years and was shared within groups online, where he was an active member (Am and Weimann, 2020; Baele et al., 2023). During his attack, his beliefs and ideology were fully absolute and binary, leaving no room for interpretation nor grey areas, evident through statements such as “*Any invader you kill, of any age, is one less enemy your children will have to face. Would you rather do the killing or leave it to your children? Your grandchildren?”* (Tarrant, 2019, p. 22). During his attack, he was cheered on through a Facebook livestream of his attack by fellow believers via 8Chan (Pratt, 2020). Mirroring the other two case studies, Brenton’s beliefs were marked by outgroup ‘othering’, perceived outgroup threat and violence-condoning norms.

- Outgroup ‘othering’: Tarrant referred to Muslim immigrants as “invaders” and “enemies.” His manifesto contains a whole section about the rape of European women by invaders and he wrote that his ultimate goal was to protect the existence of white people and children (Tarrant, 2019, p. 5–7). He also described his hatred for Westerners who converted to Islam: “*The only Muslims I truly hate is the convert, those from our own people that turn their backs on their heritage, turn their backs on their cultures, turn their back on their traditions and became blood traitors to their own race. These I hate*.” (Tarrant, 2019, p. 12).
- Perceived outgroup threat: Tarrant’s manifesto was strongly focused on the idea of a “great replacement” and “white genocide.” He repeatedly warned of the alleged ethnic, cultural and racial eradication of white people of European descent by non-white migrants. For example, he wrote: “*Mass immigration will disenfranchise us, subvert our nations, destroy our communities, destroy our ethnic binds, destroy our cultures, destroy our peoples*” (Tarrant, 2019, p. 4).
- Violence-condoning norms: In his manifesto, he called for the killing of “high profile enemies,” such as Angela Merkel, Recep Tayyip Erdoğan and Sadiq Khan (p. 39), and various others, including anti-white CEOs, drug dealers, invaders and rapists and their families (p. 32–50). He spoke of wanting revenge for Ebba Åkerlund and for other Europeans who had lost their lives in Islamic terrorist attacks, who had lost their lives to “foreign invaders.” (Tarrant, 2019, p. 5). His beliefs and writings were underpinned by a strong endorsement of violence and the glorification of previous far-right attackers.

Identity fusion with the wider racial ingroup, as well as the narrower group of 8Chan white nationalists, played a key role in Tarrant’s writings (Ebner et al., 2022a, 2024). In his manifesto (2019), he stated, “*Support your brother nations*” (p. 55) and “*I have only had brief contact with Knight Justiciar Breivik*, *receiving a blessing for my mission after contacting his brother knights*” (p. 18). The final words in his manifesto read, “*Live or die, know I did it all for you: my friends, my family, my people, my culture, my race*” (Tarrant, 2019, p. 73). His grievances and life experiences were projected onto a higher ingroup level and thus became political (Tarrant, 2019; Royal Commission of Inquiry, 2020). His identity fusion was accompanied by high levels of perceived threat against his ingroup, as noted above. He frequently discussed violence against Europeans and wrote about immigrants stealing their land (Tarrant, 2019, p. 36). A report by The Royal Commission of Inquiry into the Terrorist Attack on Christchurch (2020) said: “*His* (Tarrant) *life experiences appear to have fuelled resentment, and he became radicalised, forming extreme right-wing views about people he considered a threat. Eventually, he mobilised to violence*” (Royal Commission of Inquiry, 2020, p. 11).

### Discussion

The above study used an interdisciplinary framework to examine under what circumstances pathological fixation can lead to LAGVF, leading us to present the case for a novel framework that we have dubbed Extreme Overvalued Beliefs and Identity (EOBI). We recognise that EOBs, much like obsessions and delusions, are not inherently violent, nor do they inevitably lead to violence. While pathological fixation has been shown to precede many acts of LAGFV, it alone does not predict or explain its occurrence (Meloy and Rahman, 2021). Therefore, exploring EOBs as a risk factor for LAGVF requires the investigation of its specific manifestations as well as additional variables. To address this issue, we explored interdisciplinary theories of violence and drew on decades of research on identity fusion and its known effects on violent extremism and self-sacrifice (Swann et al., 2009; Whitehouse and Lanman, 2014; Swann and Buhrmester, 2015; Whitehouse, 2018; Reese and Whitehouse, 2021).

Identity fusion—an arguably neglected variable in SPJT—may help to explain why and when pathological fixation becomes a significant risk factor for violent extremism. While previous research in forensic psychiatry and psychology has emphasised the significance of identification in SPJT (Meloy, 2018), we recommend focusing on identity fusion instead. Relying on an evidence review and three case studies of LAGFV, our preliminary findings suggest that identity fusion plays a crucial role when determining the risk of a pathological fixation shared with a group. When a fixation driven by EOBs produces identity fusion, it appears to be particularly strongly associated with violent outcomes. Moreover, three specific types of shared beliefs re-occurred across the examined cases of LAGVF and have previously been shown to be statistically significant factors in the manifestos of violent attackers: *violence condoning norms, existential threats, and outgroup othering* (Ebner et al., 2022b, 2022a, 2024). The detected markers of fixation and fusion across the three case studies are presented in Table 1.

Our review and analysis suggest that the *Extreme Overvalued Beliefs and Identity* (EOBI) framework can be a helpful tool to guide future research in addressing relevant components across disciplines. Building on evidence from social psychology, evolutionary anthropology, and computational linguistics, EOBI combines our knowledge of pathological fixation and extreme group alignment, specifically EOBs and identity fusion, to determine the risk of LAGVF. While our findings indicate that the EOB plus fusion formula can explain some acts of violent extremism, we do not argue that this is the only pathway to violent extremism. Importantly, we would expect that pathological fixation in combination with other mental health issues can sometimes lead to violent extremism in the absence of identity fusion. For example, the Isla Vista shooter Elliot Rodger displayed signs of pathological fixation driven by EOBs based on the Involuntary Celibate subculture on Reddit (Collins and Clark, 2021); however, no indicators of identity fusion were present in his manifesto (Ebner et al., 2022a). In an analysis of the Rodger manifesto, researchers identified several vulnerabilities that we also identified in the current case studies, including grievances, humiliation, and demonisation of the outgroup, in addition to absolute thinking, aligning closely with EOBs (Capelos et al., 2024). Likewise, previous studies focused on several cases of extreme pro-group violence that were characterised by high levels of identity fusion but where data about EOBs was missing and would need to be further examined (see, for example, Whitehouse et al., 2014; Whitehouse, 2018). Even though EOBs were not investigated in these studies, related factors such as specific shared beliefs in an existential threat from an outgroup and violence-condoning norms were highlighted. Furthermore, even when all of the components proposed in our model are present, a range of inhibiting factors can influence the pathway to violent extremism. This may include a lack of opportunity, finances, capability or any social, environmental or psychological protective factors.

Another limitation of this paper is that all three exploratory case studies focus on white nationalist violence within Western cultures. While this decision was made to build on previous research and locate the proposed framework within the existing literature (Rahman et al., 2016; Ebner et al., 2022a, 2024), we encourage future research to apply the current framework in a much wider range of cultural, political and religious settings. Exploring beliefs across the ideological spectrum, as well as including both violent and non-violent extremist cases, is essential to understanding the effects of the combination of factors highlighted in our proposed framework. Our analysis specifically investigated EOBs and identity fusion, but other variables identified across the three case studies included dysfunctional or difficult family relationships, social isolation, and difficulties acquiring and maintaining social and romantic relationships. The absence of fusion with family or friends – common in the general population – may make vulnerable individuals more prone to fixation on and fusion with groups espousing extreme beliefs. Lastly, all three individuals considered here were either diagnosed (Roof) with or displayed traits (Breivik and Tarrant) of Autism spectrum disorder. Studies have demonstrated links between Autism spectrum disorder and extremism, with notably higher prevalence among lone actors compared to group actors or the general population (Corner et al., 2016; Allely et al., 2024). Autism spectrum disorder is not directly linked to violent behaviour, but considering the overrepresentation in lone-actor extremist samples, associated factors and vulnerabilities warrant further investigation (Allely et al., 2017, 2024). In light of the patterns observed in the current analysis, the link between Autism spectrum disorder and fixated thinking in LAGFV could potentially be worth exploring in more depth. In the DSM-5, one of the key characteristics of Autism spectrum disorder is “*highly restricted, fixated interests that are abnormal in intensity or focus* (e.g.*, strong attachment to or preoccupation with unusual objects, excessively circumscribed or perseverative interests*)” (American Psychiatric Association, 2013, pp. 50). It is beyond the scope of the current study to consider all the psychosocial factors that may have played a role in all three cases. However, while acknowledging the selection bias present in the current review, we recommend that future research explore the link between pathological fixation, identity, autistic traits and lone-actor grievance-fuelled violence.

While our paper provides some preliminary evidence that there appears to be an important relationship between fixation in the form of EOBs and identity fusion that may help explain common forms of violent extremism, more research needs to be done to determine the exact nature of the interaction between the two factors. To confirm the relevance and relationship of both factors, quantitative analysis would be needed to test their statistical significance and possible causal effects in pathways to violence. While identity fusion has been extensively explored, fixation and EOBs would benefit from more research and a comprehensive scientific understanding. Future research that further explores extreme forms of fixation, group alignment and other potential risk factors across scientific disciplines can make a valuable contribution to creating more robust threat assessments that draw on strong theoretical underpinnings across scientific disciplines.

More effort to establish a common vocabulary and closer collaboration and information exchange across disciplines would be valuable, not only for threat assessment protocols but for the practical application of scientific knowledge across various academic disciplines. It would benefit research in forensics, medicine, psychology and social sciences to establish bridges between overlapping theories and research. As theoretically driven approaches have been secondary to data-driven approaches in threat assessment (Gill and Rottweiler, 2023) and solely focusing on idiosyncratic beliefs and mental illness is not sufficient, we strongly believe in the benefits of interdisciplinary approaches to explain violent behaviour. For example, future studies should focus on relational ties that may emerge even in the absence of direct relations, i.e., in the form of extended fusion, which likely plays a determining role for EOBs (Swann et al., 2012), as well as research on sacred values, which would be exceptionally relevant for fixation and EOB (Atran and Axelrod, 2008; Whitehouse, 2018). Applied collaborative approaches to improve forensic evaluations and decision-making could benefit current clinical conceptualisations that require further theory-driven support.

To conclude, our EOBI framework combines research on EOBs as a driver for fixation with decades of research on identity fusion. We do not argue that fixation or identity fusion are *necessary* or sufficient predictors of violent extremism. However, we believe that a combination of the two factors is commonly observable in pathways to extreme pro-group violence, particularly when fixation focuses on a combination of outgroup threat, othering, and violence-condoning norms. While we acknowledge the limitations of our proposed EOBI framework and the need for future research to further test and refine the framework, our analysis indicates that the highlighted risk factors may increase the risk of violent extremism. Importantly, identity is a neglected variable in the context of fixation, especially when shared with others. The threats posed by online subcultures remain a complex and multifaceted issue that requires strong theoretical and data-driven approaches to address. Using diverse methodologies, empirical data and larger datasets, future interdisciplinary research could improve current understanding and conceptualisations for prevention and intervention strategies.

## Footnotes

^1^In this paper, we will use “Violent Extremism” as an umbrella term to capture the full spectrum of lone-actor and group-based acts of violence. As we will describe in more detail in the section “Identification or Identity Fusion?” our focus is on behavioural rather than ideological aspects of extremism.

^2^It is important to note in this context that the coexistence of fixation drivers is a possibility and has been observed in previous cases (Kupper et al., 2023). However, the focus here will be on the concluding notion of competency, fixation driven by EOB, and growing deterioration leading to fusion and violent extremism.
